# Mechanism and clinical evidence of immunotherapy in allergic rhinitis

**DOI:** 10.3389/falgy.2023.1217388

**Published:** 2023-08-01

**Authors:** Marco De Carli, Eleonora Capezzali, Silvia Tonon, Barbara Frossi

**Affiliations:** ^1^ Second Unit of Internal Medicine, University Hospital of Udine, Udine, Italy; ^2^ Department of Medicine, University of Udine, Udine, Italy

**Keywords:** allergic rhinitis, allergic immunotherapy, gender differences, rhinosinusitis, vaccines

## Abstract

Allergic rhinitis is a common upper airway disease caused by hypersensitivity to various aeroallergens. It causes increased inflammation throughout the body and may be complicated by other otolaryngological pathologies such as chronic hyperplastic eosinophilic sinusitis, nasal polyposis, and serous otitis media. Allergic rhinitis is an IgE-mediated disease and immunotherapy can be a possible approach for patients to limit the use of antihistamines and corticosteroids. There is evidence that allergen immunotherapy can prevent the development of new sensitizations and reduce the risk of later development of asthma in patients with allergic rhinitis. However, some patients do not benefit from this approach and the efficacy of immunotherapy in reducing the severity and relapse of symptoms is still a matter of debate. This review highlights new aspects of allergic rhinitis with a particular focus on the impact of sexual dimorphism on the disease manifestation and efficacy to the allergen specific immunotherapy.

## 1. Introduction

Allergic rhinitis (AR) is an inflammation of the nasal mucosa characterized by sneezing, itchiness, rhinorrhea and nasal obstruction, caused by hypersensitivity to inhaled allergens. The symptoms of AR may be variegated and arise after inhalation and primary sensitization to few or numerous allergens such as pollen of various plants, house dust mite, hair, and organic material from certain animals (1, 2).

AR is estimated to affect around the 20% of the occidental world population causing a detrimental effect on productivity and lifestyle quality, including the development of emotional challenges and a decline in sleep, social interaction, daily activities, and work and academic performance (3, 4). In fact, AR is the most prevalent cause of absenteeism and/or presenteeism at workplace, and the global loss of job productivity caused by AR is noticeably greater than that caused by other common pathologies including depression, infections, diabetes (5). Comorbidities of the upper and lower airways, such as rhinosinusitis and asthma, can worsen AR and may be a factor in the observed impact on productivity and quality of life (6). These often mild and not too much disabling symptoms should not be ignored from patients, clinicians and researchers considering the numerous evidences about AR as a significant risk factor for asthma onset shown now more than 20 years ago (7), and maintained strongly valid from the ARIA (AR and Its Impact on Asthma) initiative, nowadays (8).

In this review we update on relevant topics on AR, in particular on gender-related differences in the prevalence and manifestation of AR and the molecular mechanism behind them. Moreover, we review the current knowledge regarding the use of allergen specific immunotherapy (AIT) for the control of AR especially when AR is associated with asthma or chronic rhinosinusitis.

## 2. Allergic rhinitis: manifestation and genetics

AR can be phenotypically classified following several criteria such as severity (mild, moderate, or severe), time and type of exposure (seasonal or perennial), as well as duration of symptoms (intermittent or persistent) (9). Moreover, in the last decades, AR has been classified into two additional classes: occupational, associated with allergen possibly present in the workplace, and local, with nasal evidence of IgE production in absence of systemic atopy (10, 11).

AR is often associated to other comorbidities of the airways. Indeed, AR and asthma are strongly correlated also because they show a similar immune response for what concerns inflammatory cells and mediators even if they differ in the extent of the immune response itself. As a proof, a metanalysis of European and non-European studies confirmed that history of AR is greatly linked to asthma even if with different association in different studies (12). Rhinitis can be also associated with conjunctivitis, which is always of allergic origin (allergic rhinoconjunctivitis); in such case, there are symptoms affecting the eyes, including: tearing, redness and burning of the eyes, intolerance to light (photophobia) and foreign body sensation. Moreover, AR is considered a comorbidity factor in several otolaryngological disorders and is usually thought to be a key factor in the development of chronic rhinosinusitis, although their association is still poorly understood and remain controversial.

Regarding the cellular and molecular events causing AR manifestations following antigen exposure, both elements of innate and adaptive immunity, including mast cells, macrophages, eosinophils, B lymphocytes and CD4^+^T lymphocytes, intervene in the response (13). The immune response after allergen exposure can be divided into two distinct phases: an immediate early phase and a delayed late phase. In the early phase, after allergen exposure, antigen presenting cells (APCs) expose allergen peptide on their surface through major histocompatibility complex (MHC) class II molecules, move to lymph nodes, and induce differentiation of naïve CD4^+^ T cells into specific T helper 2 (Th2) cells. Then, these cells produce IL-4 and IL-13 which in turn allow B cell isotype-switching to IgE production. IgEs are then recognized by specific cell-surface receptors (FcεRI) on mast cells, for further release of proinflammatory mediators (such as histamine, prostaglandin, leukotrienes, TNF-α) mainly through degranulation. In the late phase, so 4–6 h from allergen encounter, different cell types (such as T cells, granulocytes, and monocytes) as well as numerous molecular mediators (such as elastase and basic proteins) are involved (14, 15).

In the recent years, several genetic and epigenetic analysis of genes encoding molecules possibly involved in the pathogenesis of AR have been conducted and published (16, 17). There are reports of polymorphisms in genes encoding chemokines or chemokines receptors (CCR1, CCR2, CCR5, CCXCR1, SDAD1, CXCL9, CXCL11, CSCL10, RANTES, and eotaxin-3), interleukins or their receptors (IL-13, IL-18, IL-21, IL-27, and IL-23R, IL-12RB1, IL-28RA) molecules involved in cell signaling (GATA), as well as molecules involved in Th2 responses such as FcεRI and leukotrienes (e.g., LTC4S). Studies of classical HLA alleles and amino acid variants, identified HLA-B and HLA-DQB1 as the strongest associated HLA class genes to AR [reviewed in reference (16)] In the 2018 a genome-wide association study carried out a large-scale meta-analysis of 16,531,985 genetic markers from 18 studies comprising 59,762 cases and 152,358 controls discovering 20 novel loci (18). Most of these novel loci have functions in innate and adaptive immune processes and include IL7, SH2B3, CEBPA-CEBPG, CXCR5, FCER1G, LTK, NFKB1, and TNFSF 11 genes (18).

Concerning the role of epigenetics in AR, a methylation profile in nasal epithelium was recently found to associate with AR (19). The genome-wide DNA methylation study performed by Qi et al. identified CpG sites significantly associated to AR and demonstrated that methylation of the cg03565274 sequence was negatively associated with AR, but positively associated with having a pet, cat or dog, at home during childhood and with protection from developing asthma and/or AR (19). In the same study DNA methylation was related to gene transcripts that are expressed in immune and epithelial cells and/or involved in immune pathways and was mainly driven by specific IgE–positive subjects. Another recent study supported that DNA methylation is associated with IgE sensitization early in life and provided evidence that maternal DNA methylation levels are associated with IgE sensitization in the child supporting early *in utero* effects on atopy predisposition (20).

## 3. Gender differences in allergic rhinitis

**Figure 1:**
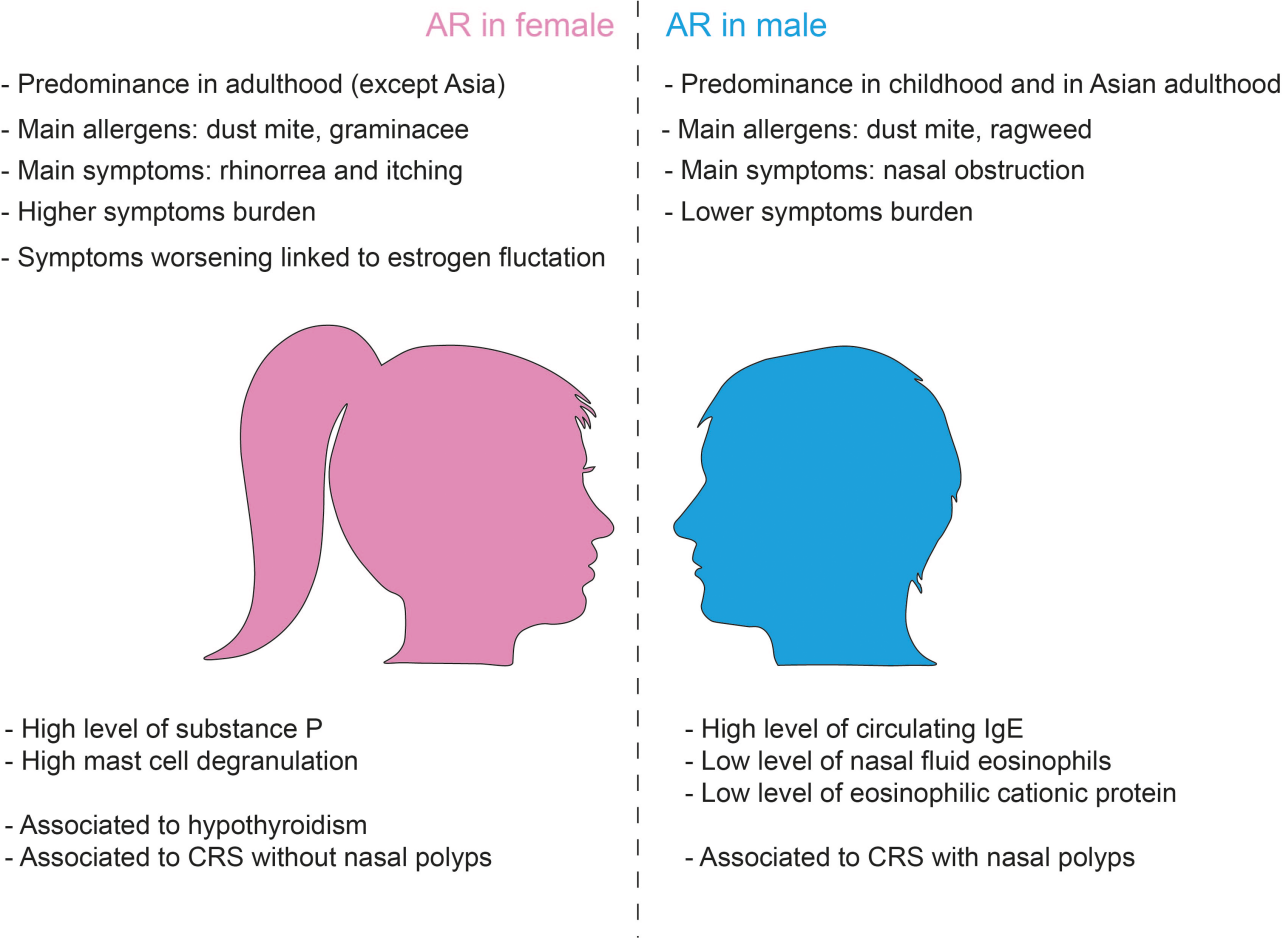
Gender differences in allergic rhinitis.

Over the past decades, a great body of literature has documented the differences in prevalence and severity of allergic disease between men and women (21), especially respiratory allergies with generally a male predominance during childhood that shifts to female predominance in adolescence and adulthood (22) (Figure 1).

Regarding gender differences in AR, the studies conducted so far have led to controversial conclusions, even if in general they seem to indicate a higher prevalence of AR in the female population (21, 22). In a Spanish study on 428 adult rhinitis patients randomly selected from a population evaluated for the first time for rhinitis over 1 year, the majority were <30-year-old, nonsmoking, with no family history of atopy, and severe or moderate persistent and perennial rhinitis affected women (11).

The most vulnerable allergic women are those with greater cycle irregularities, because these further increase hormonal fluctuations. This has been widely addressed in asthmatic women (23) and it is suggested to be possible true also for AR female patients but at the moment evidences are still vague (24). However, a substantially higher risk of allergic rhinitis was also observed in women who experienced first menstruation at a younger age (25). Finally, longer duration of reproductive period was associated with higher prevalence of AR and aeroallergen sensitization in the postmenopausal period (26).

Conversely, a 12-years retrospective study of Hong and colleagues analyzed by sex and age distribution about 2,000 patients with nasal symptoms that underwent a skin prick test with standardized allergens and let to different conclusions (27): the prevalence of AR is higher among boys than girls during childhood (0–10 years), while females show a higher prevalence during adolescence (11–17 years) as compared to their male counterparts. However, the authors reported a the prevalence of allergen sensitization peaks at between 20 and 29 years that progressively decreases reaching no difference in prevalence between genders in the adult age. Notably, the sensitization rate to allergens differs depending on the type of specific allergen considered. For example, in adulthood (age >60 years) the sensitization rate to house dust mites decreases with age, while sensitization to mugwort and ragweed increases (27). Interestingly, in childhood the prevalence of sensitization to house dust mites is more frequent in male children compared to female with older children (>6-years) more sensitive than younger (2–5 years old). Moreover, sex related differences in sensitization to pollens have been reported: late spring flowers were found to be the most common allergen to which female were sensitive while summer autumn flowers allergens were common in male, but the reason is unknown (28). Nasal obstruction tends to be more prevalent in males than in females, while rhinorrhea and itching seem predominant in females (27). Interestingly, total nasal symptom scores did not differ by sex (27).

Another recent systematic review and meta-analysis conducted on 6,539 publications, including overall 291,726 males and 301,781 female AR patients, demonstrates that among children, significantly more males than females have rhinitis symptoms, while an opposite trend is observed in the case of adolescents aged 11–18 years. Intriguingly, these findings were steady worldwide except for Asia, where a male predominance persisting beyond childhood was noticed. In adulthood, no prevalence in either males or females was found, but the number of studies was lower compared with those on young patients (29). All these data suggest that there is a sex-related difference in AR prevalence switching from male to female predominance around puberty. Nevertheless, longitudinal studies with follow-up prolonged into adulthood are mandatory to obtain definite data about the influence of sex on AR.

The mechanism underlying differences in allergy manifestation between woman and man can be the results of sex-specific genetic differences related to X chromosome and on the effect of sexual hormones on the modulation of the immune system responses and inflammation that change from puberty to adult age (Figure 1).

Classically, allergic reactions are Th2 inflammatory reactions mediated by CD4^+^ Th cells: several reports demonstrate that adult females have increased Th2 response with higher levels of IL-4 and IL-13 expression than males (22). However, even if women are found to experience a more severe burden than men, they are more likely to have lower amounts of nasal fluid eosinophils and eosinophilic cationic proteins and lower levels of nasal fluid IgE, despite having reactions similar to men (30). Gene expression analysis of allergen challenged CD4^+^ T cells from patients with AR showed downregulation of signaling pathways that regulate chemotaxis in females suggesting gender differences in specific inflammatory mechanisms in AR patients (31). In this study, Barrenäs and colleagues identified ten inflammatory pathways that were differently expressed between men and women, five of which have reduced expression in women. The downregulated genes included key factors involved in chemotaxis and in particular CCL4, whose levels in the nasal fluids of women were found to be lower than in men (31). However, more recently, Tomljenovic and colleagues demonstrated that under resting condition female patients with seasonal AR show higher levels of circulating substance *P* than males and suggested that this difference could cause a stronger neurogenic reaction in women (32). Indeed, in response to both allergen-specific and non-specific nasal challenge, female patients experience a stronger burning sensation with substance *P* that is significantly increased compared to men (32).

Moreover, fluctuations in estrogen levels, at ovulation, but perhaps above all the fall in estrogen and progesterone levels before menstruation, may also be responsible for worsening of AR and asthma symptoms. In fact, estrogen increase the reactivity of both innate and adaptive immune cells—mast cells, but also eosinophils- and lead them to release great quantity of substances that cause inflammation and bronchoconstriction into the tissues. Several studies have demonstrated that estradiol affects the extent of mast cell degranulation during IgE-mediated response (33). More in deep, estrogen stimulation enhances the adhesion of eosinophils to mucosal microvascular endothelial cells, whereas their stimulation by the combination of estrogens and progesterone induces degranulation (34). In the case of mast cells and basophils, it has been clearly shown that these cells mature and degranulate upon stimulation with estrogens (35). In fact, *in vitro* data shows that preincubation of basophils or mast cells with physiological concentrations of estrogens increase the subsequent histamine release induced by cross-linking of FcεRI (36, 37). Estrogens can increase rhinitis symptoms even by acting directly on parasympathetic system which in turn inhibits acetylcholinesterase activity resulting in acetylcholine increased levels (38) and boosting the expression of H1 histamine receptor mRNA in nasal epithelial cells (39). Moreover, estrogens can affect the nasal mucosa inducing interepithelial edema, glandular hyperplasia, fibrous tissue deposition and new vessel formation (40). Therefore, not surprisingly, the incidence of vasomotor rhinitis occurs in about 20% of pregnancies and is particularly present in the last trimester, when estrogen levels are relatively high. Rhinitis itself is also associated to other conditions with elevated estrogen levels, such as puberty, menarche as well as during estrogen replacement therapy and oral contraceptive use (41, 42).

Besides estrogen and progesterone, thyroid function has a role in modulating nasal symptoms, too. It has been reported that hypothyroidism can lead to a hormonal rhinopathy that is characterized by prolonged mucociliary clearance with increased risk of upper respiratory and sinonasal infections (43). Furthermore, thyroid replacement therapy is associated with clinically significant improvement in turbinate hypertrophy, mucosal pallor, clearance time and nasal peak flow in hypothyroid subjects with allergic rhinitis (40) and it has been shown that hypothyroidism has a higher incidence in AR patients (44). Intriguingly, hypothyroidism and Hashimoto's disease are both more common in women than in men with a 10/1 male female ratio in the case of Hashimoto's thyroiditis (45, 46). This may be a further factor able to influence a differential expression and severity of rhinitis symptoms in female.

## 4. Pharmacological treatment of patients with allergic rhinitis

The treatment of choice for AR should be the elimination of the environmental allergen responsible for the symptoms. However, despite this recommendation, complete allergen control is extremely difficult to achieve and scientific evidence to support allergen prevention measures is limited while an effective management of the disease can be achieved through a comprehensive approach combining prevention, allergen control and pharmacological treatment (1, 8).

The most common first-line treatment for AR are antihistamines that are particularly effective in reducing nasal itching, sneezing, and nasal discharge, while antileukotrienes exert a predominantly anti-inflammatory effect. Compared to first- and second-generation H1-antihistamines, with sedating and cardiotoxic side effects, the third-generation of oral antihistamines, such as desloratadine, fexofenadine and levocetirizine, have improved safety and effectiveness in controlling AR symptoms (47).

Leukotriene (LT) receptor antagonists, such as montelukast and zafirlukast (48), bind to the cysteinyl LT receptors and block the ability of cysteinyl LT of promoting inflammation, mucus production and nasal congestion. LT antagonists were more effective than H1 antihistamines for symptoms throughout the night but not for symptoms during the day, according to meta-analysis research (49).

Through their agonistic activity at 1 and 2-adrenergic receptors on nasal mucosal endothelial cells, nasal decongestants alleviate nasal congestion symptoms by reducing mucosal edema. Commonly used nasal sprays are oxymetazoline, phenylephrine, and pseudoephedrine. However, if excessively used nasal decongestant might result in rhinitis medicamentosa (i.e., a situation in which rebound blockage occurs after ceasing nasal decongestants) (50), which is treated by administering intranasal corticosteroid.

Intranasal corticosteroids are useful for treating both mild and moderate-severe AR in both children and adults and represent a non-specific symptomatic treatment as they inhibit immune cells. For severe or uncontrollable symptoms, systemic corticosteroids (by mouth or by injection) should only be used as a last choice.

Mast cell stabilizers, such as sodium cromoglicate, interfere with exocytosis of mast cell granules and can limit the release of inflammatory mediators from activated mast cells (51). Consequently, cromolyns, administered as nasal sprays, should be used immediately before exposure to the allergen, for a protective effect. They are relatively safe drugs however, their short half-life and inferior efficacy compared to other drugs limit their effectiveness in treating AR (52).

An adjunct to symptomatic drug treatment, nasal irrigation with topical nasal sprays containing isotonic sodium chloride solution is used.

## 5. Allergen specific immunotherapy

Since some patients with AR do not benefit from standard medical care, allergen specific immunotherapy (AIT) could be employed as a disease modifying therapeutic approach.

AIT was first reported by Noon (53) and Freeman (54) through the inoculation of grass pollen extracts in patients with seasonal AR, before the pollen season, resulting in desensitization and reduction of symptoms occurrence after allergen exposure (55).

AIT involves the repeated administration of high-dose allergens towards two main ways: subcutaneous immunotherapy (SCIT) or sublingual immunotherapy (SLIT) for at least 3 years to confer clinical benefits (56). Intralymphatic, epicutaneous and local nasal administration of immunotherapy have been also investigated: among them, intralymphatic immunotherapy (ILIT) has been proposed as a faster, more efficient, safer, and lower cost approach (57). It consists in three monthly ultrasound-guided injections with allergen over 8 weeks. A recent randomized double-blind placebo-controlled clinical trial has shown that 2 years after ILIT, the actively treated group reported significantly fewer symptoms, lower medication use and improved quality of life. Clinical improvement is associated with immunological changes such as increased Treg frequencies and grass-induced IFN-γ production (58).

Allergen formulation employed for conventional immunotherapy varies among different studies. In many cases, allergen extracts for immunotherapy arise from the whole extracts rather than the major allergen. Mono and poly-allergen AIT are performed by choosing the specific allergens responsible for the symptoms. Poly-allergen AIT may be administered simultaneously or at different times (59). Similar effectiveness has been demonstrated by AIT protocol based on purified major allergens and chemically modified major allergens. For example, AIT with natural purified major birch pollen allergen, Bet v 1 or recombinant Bet v 1 were demonstrated to be as effective as AIT with birch pollen allergen extract (60). Similarly, the purified major allergen of *Alternaria alternata*, Alt a 1, caused reductions in the allergic symptoms scores in AR patients (60).

Advances in molecular cell biology have enabled the use of recombinant wild type allergens, recombinant hypoallergens (which, by DNA technology, convert allergens to abolish IgE activity but leave the T–cell response), and recombinant fusion proteins (carrier proteins and non-allergenic allergen-derived peptides that contain tolerogenic epitopes) (61). Recombinant allergen-based vaccines that use allergen-encoding DNA have also been developed for both SCIT and SLIT. The aim is to reduce IgE response and to increase production of blocking allergen-specific IgG antibodies. To improve the efficacy of recombinant hypoallergens, recombinant vaccines containing B cell epitopes have been developed (62). These vaccines don't possess allergenic activity, inhibited allergen-specific T-cell responses but showed high immunogenicity throughout induction of IgG responses by the carrier protein and thus had high efficacy with less adverse effects (62). BM21, a vaccine for grass pollen, is another example of recombinant fusion protein-based vaccine. It contains non-IgE-reactive peptides derived from the IgE-binding sites of the grass pollen allergens and is covalently linked to Pre S from hepatitis B virus, a viral protein carrier that provides carrier-specific T cell help (63). The vaccine was tested in multi-center, double-blinded, placebo-controlled study and was demonstrated to relieve allergy symptoms without severe adverse effects (64).

Notably, the identification of the specific allergen is mandatory. Molecular diagnosis of allergy is constantly evolving and has been implemented as a complementary diagnostic tool for AIT (65). To better improve patient selection for AIT, more effort has been dedicated on the characterization of allergen extracts using transcriptomics and proteomics approaches and profiling of their IgE reactivity (66, 67). Moreover, component-resolved diagnostics has been brought to identify sensitization to allergenic proteins and to select patients in order to improve AIT efficacy in polysensitized patients (68). In a Spanish study conducted on patients with seasonal AR to grass and olive pollens that were addressed to AIT basing on skin prick tests or on the indication from the component-resolved diagnostics, it has been demonstrated that AIT prescribed on the basis of second methods was more accurate and reduced the cost of immunotherapy (69).

## 6. Molecular mechanism of allergen immunotherapy in the treatment of AR

AIT is based on the long term and repeated administration of specific doses of allergens to modify the immune response and induce protective immunity. The administration of AIT achieves a successful outcome when the allergic patient develops tolerance to the allergen. Specifically, AIT is reputed to be working not only when the patient lives his or her life without discomfort caused by the symptomatology, but also when from an immunological point of view a tolerance profile is achieved in the context of both innate and adaptive immunity. Indeed, AIT uses general mechanisms of immune tolerance to allergens to normalize allergen-specific T and B cells, IgE and IgG production, as well as modification of mast cells and basophil activation thresholds. The efficacy of AIT depends on one side on the induction of allergen-neutralizing IgG antibodies that block allergens at the mucosal sites, preventing FcεRII-mediated facilitated allergen presentation, and inhibiting mast cell degranulation and allergic inflammation. On the other side it depends on maintenance of regulatory cells (T and B cells, regulatory B cells, and various other myeloid regulatory cells) in order to suppress type 2 immune responses and allergic inflammation.

Treatment for AR that focuses on immune regulation aims to shift the normal courses of response to an allergen rather than bringing about a transformation to an immunologically ignorant or unresponsive state.

### 6.1. T cell response following AIT

Part of the success of AIT is the unresponsiveness of T cells to the allergen. This occurs in presence of anergic T cells possibly due to high-dose tolerance (70) and a reduction in proliferative response via IL-10 (71).

In the context of subcutaneous AIT (SCIT) there is evidence of a local increase in the tolerogenic CD25^+^ and Forkhead box P3 (FOXP3^+^) Treg population in the nasal mucosa of treated patients (72). Besides that, in sublingual AIT (SLIT), epigenetic modifications at the FOXP3 promoter level can be observed, leading to a sustained production of Tregs and their suppressive function (73, 74).

As mentioned above, a key role in the context of AIT is played by IL-10 which, following therapy, is produced in high amounts by T lymphocytes along with other suppressive cytokines (such as TGF-β and IL-35), driving the generation of a tolerogenic immune phenotype: Treg1 cell proliferation, inhibition of Th2 response and, more in general, reduction of tissue-infiltrating proinflammatory cells (75).

Beyond cytokine production, Tregs control suppressive mechanism thanks to metabolic pathways disruption, surface molecules capable of blocking dendritic cells activation, and release of cytotoxic molecules (76).

### 6.2. B cell response following AIT

After AIT, B cells tend to assume a more protective role modifying their phenotype in terms of type of antibody and cytokine produced, inducing immune tolerance directly or indirectly in the presence of the allergen.

For example, regarding antibody production, an isotype switch is observed: instead of allergen specific IgE, IgG4-blocking antibodies against the same antigen are produced (77). In addition to this, as far as it concerns cytokine production, a particular subset of B cells, the regulatory one (Breg), produce high amounts of the anti-inflammatory cytokine IL-10, which reduces antigen presentation by APCs and prevents Th2 from secreting IL-4, which is capable of stimulating IgE production (78). IL-10 activity represents an additional stimulus to the production of IgG4 instead of IgE, leading to a significative improvement in the allergic patient's symptoms (79, 80).

### 6.3. Innate immune cell response following AIT

It has been shown that following AIT, mast cells and basophils, which play a pivotal role in initiating the allergic response, undergo rapid desensitization resulting in a dropped reaction to the allergen even in presence of high levels of specific IgE. At longer times, a decrease in tissue infiltration and release of proinflammatory mediators as well as reduction of allergy-associated receptors are observed (81, 82). However, most of the mechanisms leading to this suppression are not fully understood.

Following AIT administration, the produced IgG inhibit IgE-mediated degranulation through FcεRI disaggregation, with a simultaneous increase in low-affinity FcγRII exposure on circulating basophil surface: in particular, IgG3 and, to a lesser extent, IgG2 appear to play this role (83, 84).

Another cell population of innate immunity that plays a crucial role in AIT response is that of innate lymphoid cells (ILCs). Of these cells, those belonging to group 2 (ILC2) are the ones that most seem to be involved in allergic rhinitis context and show differences in their phenotype following immunotherapy administration. More specifically, what is observed is a significant decrease in ILC2 frequency (85) accompanied by a switch from a proinflammatory phenotype (in terms of cytokine production) to a more tolerogenic one (86). Contextually, a recent randomized clinical trial has allowed a better characterization of the acquisition of IL-10-producing capacity by ILC2s upon activation with IL-33 and retinoic acid, leading to an important contribution to immunogenic tolerance to aeroallergens, following AIT administration (87).

## 7. Effectiveness of allergic immunotherapy

AIT is to date the only established treatment able to modify the IgE-mediated allergic diseases by treating the underlying immunological mechanism (88). Most of the studies in the past have consistently shown that AIT is effective in lowering symptoms and medication use (89, 90). It has also been reported a possible effect of AIT in preventing asthma development and the onset of new allergen sensitization (88, 91, 92). Some studies also demonstrated a lower prevalence of allergy in children born to mothers who underwent AIT during pregnancy (93).

However, many meta-analysis and reviews outlined that major limitation in evaluating the clinical outcome of AIT are the broad diversity in composition of products, the study designs, the heterogeneity of the environmental conditions, the age of the population involved and the use of different primary and secondary study endpoints (88, 94, 95). Moreover, different outcomes have been reported between SLIT and SCIT, as well as between SLIT delivered by tablets or by drops (88).

In order to assess the real-world and long-term effectiveness of AIT in the treatment of AR in a broad population, the retrospective REAl-world effeCtiveness in allergy immunotherapy (REACT) study investigated a score-matched cohort of studies published from 2007 to 2017. The study included almost 100,000 (subjects matched 1:1 with controls) and indicated the overall efficacy of AIT in improvement of clinical and symptoms in AR patients with AIT prescription compared to patients with no AIT prescription across the 9 years of follow-up (96). The following subgroup analysis of REACT study performed by Contoli et al. evaluated the effectiveness of AIT in AR according to the route of administration, type of allergen and persistence of AIT treatment. In this study SCIT and SLIT approaches showed similarly greater reductions in AR prescriptions than controls. Comparably greater reductions were observed for grass and house dust mite specific AIT than for controls, but significantly smaller reductions were observed for tree-specific AIT (97).

However, the magnitude of the clinical efficacy of AIT remains difficult to establish, due to the use of different types of allergen extracts, doses, dosing regimens and evaluation period (98, 99). Indeed, standardized extract dose and clinical data are not available for all allergens and the extracts used for AIT in each country have different potency, contain different allergen concentration, allergen mixtures, and adjuvants. The heterogeneity among the results seen in the different studies point out the need of a standardization of clinical endpoints (94). At today, the EAACI and ARIA guidelines recommend considering AIT in patients with AR and evidence of IgE sensitization who present with moderate to severe symptoms that interfere with normal daily activities or sleep, despite regular pharmacotherapy and appropriate strategy allergen avoidance (100, 101).

Finally, despite reported sex- and gender- related differences in AR, to our knowledge no data on differences in response to AIT between male and female AR patients have been produced so far. There are some evidences that male subjects were more likely to be non-adherent than female subjects to AIT (102, 103) and this would indirectly suggested that AIT could be more effective in the female population. But further studies to prove this hypothesis are required.

## 8. Association between allergic rhinitis and other allergic airway comorbidities: does immunotherapy represent an efficacious approach?

### 8.1. AR and asthma

Patients with AR frequently suffer simultaneously of asthma sharing the same trigger factors, including pollens, house dust mite, pets, mold, and similar pathogenetic mechanisms. About 10%–40% of patients with AR have also allergic asthma as well as 60%–80% of asthmatic patients experience AR (101) and the their co-existence enhances the overall disease burden (104). Therefore, patients with AR associated to asthma should ideally benefit from AIT.

Indeed, some cohorts showed a benefit of AIT in preventing the onset of asthma in AR patients (105, 106). Randomized controlled trials have confirmed the efficacy of AIT in patients with comorbid asthma caused by house dust mite, grass and pollens, particularly of SLIT-tablet (107).

The EAACI guideline recommended AIT as an add-on to regular asthma therapy in adults with controlled or partially-controlled house dust mite driven allergic asthma (108), in which controlled asthma is defined as daytime symptoms <2 times/week, no night awakenings, relief is needed for symptoms <2 times/week, and no activity limitation due to asthma. “Partially-controlled asthma” is defined as failure to meet the first 2 criteria above.

Moreover, the REACT study (96) demonstrated the overall efficacy of AIT in patient with AR and concurrent asthma with a consistent reduction in AR and asthma symptoms, asthma exacerbations and hospitalization (96).

### 8.2. AR and chronic rhinosinusitis

AR is considered a comorbidity factor in several otolaryngological disorders and is usually thought to be a key factor in the developing of chronic rhinosinusitis (CRS). Chronic rhinosinusitis (CRS) is defined as an inflammatory condition of the nose and paranasal sinus, characterized by nasal discharge, nasal obstruction, hyposmia or anosmia, and facial pressure that lasts more than 12 weeks (109).

CRS is classically divided into two clinical phenotypes based on the presence of endoscopically visualized polyps in the middle nasal meatus: CRS with nasal polyps (CRSwNP) and CRS without nasal polyps (CRSsNP). Novel classification criteria have been recently introduced to identify other endotypes of CRS based on the anatomic distribution (localized or diffuse disease), endotype dominance (understanding the underlying pathophysiology in association with raised IgE, IL-5, eosinophilia, and periostin), and clinical phenotypes (110). Primary CRS is categorized into localized (typically unilateral) and diffuse (not limited by functional sinonasal units or spaces). Among localized primary CRS, two phenotypes are distinguished: allergic fungal rhinosinusitis (AFRS) and isolated sinusitis; while clinically diffuse primary CRS are subdivided into eosinophilic chronic rhinosinusitis (eCRS), AFRS and central compartment allergic disease (CCAD) or non-eosinophilic chronic rhinosinusitis (non-eCRS) (110).

While paranasal sinuses are of similar size at birth, they become larger in males leading to differences in ostium location and presumably to different susceptibility to develop CRS related. In fact, CRS and CRSsNP are more prevalent in females while CRSwNP is more prevalent in males (111). CRS symptom burden is higher in females before and after endoscopic sinus surgery; however, there are no difference in endoscopic sinus surgery between sexes (111).

The pathogenesis of the CRS is not completely understood; however it is likely that allergy-induced inflammation of the sinus mucosa can lead to local obstruction and predispose to infection leading to a chronic state of the disease. It has been recently demonstrated that CRSwNP is characterized by local production of polyclonal IgE idiotypes (112). These IgE can promote proallergic inflammation and could be partially antagonized by corresponding IgG idiotypes (112) suggesting a potential benefit of AIT in patients with CRSwNP. However, the scientific literature produced so far has not proved sufficient evidence.

In 2004, the group of Nathan and colleagues developed a questionnaire to evaluate the impact of immunotherapy in the treatment of 114 atopic patients with associated sinusitis and published that 99% of patients had benefit from the immunotherapy, with a mean reduction of around 50% of symptoms, suggesting that immunotherapy could be effective in treatment for patients with AR and sinus disease (113).

A systematic review published in 2014 (114) analyzed 7 studies and assessed the efficacy of immunotherapy on clinical outcomes of different types of patients with CRS (with and without polyps, and allergic fungal rhinosinusitis subgroups). Compared with untreated patients, the ones treated with immunotherapy showed reduction of symptoms in the short-term, improvements in radiographic assessments, as well as decreased necessity for revision surgery, interventional office visits, and intranasal and oral steroid use. However, none of the studies were randomized controlled trials and conclusions were limited by the paucity of available data.

As concerning AFRS, a subtype of Th2 chronic rhinosinusitis whose determination criteria include also type 1 hypersensitivity to fungi confirmed by skin test or serum-specific blood test positivity, conclusive evidences of efficacy of immunotherapy are not present to date. Indeed some studies demonstrated significant improvement in the endoscopic disease score and CRS survey symptom score, and decreased systemic corticosteroid use in patients with AFRS after allergic immunotherapy treatment (115, 116). Other studies fail to demonstrate a significant benefit for allergic immunotherapy (117).

In a recent review published in 2020, it has been stated that intranasal allergens may not penetrate the paranasal sinuses, but instead exert their effects indirectly by means of downstream, systemic factors that then feedback to the sinuses. The authors concluded that there is only limited evidence linking AR and CRS, but some subtypes of disease such as CRSwNP and AFRS may be most related to AR (118). This was also confirmed by another study conducted in 2020 by Herych and colleagues on 90 patients affected by chronic polypus rhinosinusitis in combination with fungal sensitizations. The authors evaluated the effect of various treatment options on the clinical course of the disease in these patients and conclude that AIT leads to significant improvement of clinical symptoms (119).

## 9. Conclusions

There are several evidences supporting that AIT alleviates symptoms, reduces medication requirements and improves the quality of life in AR individuals. AIT applied in the early stage of allergic disease seems to have a good preventive effect on progression of AR to asthma, especially in young children and in the development of new sensitizations. Female populations who appear to suffer more from AR symptoms in adulthood, may benefit more than male counterpart from AIT, but more studies must be conducted to confirm this hypothesis.

As concerning association of AR and chronic rhinosinusitis, it even remains controversial. Despite being a recommendation for patients, the benefit of immunotherapy in the management of CRS is uncertain and it should only be considered as on option.

However, AIT still shows high degree of heterogenicity and effectiveness that depend on multiple factors, including the allergen dose, the presence or the absence and the type of adjuvant, the way of delivery (subcutaneous or sublingual), treatment duration, and individual immunological response.

In conclusion, beyond the advances in diagnosis and development of novel vaccine formulations, and beyond various evidences of AIT efficacy in AR, a standardization of the composition of products as well as technological improvements are needed to further increase the efficacy of AIT.
